# A secondary analysis of the childhood obesity prevention Cochrane Review through a wider determinants of health lens: implications for research funders, researchers, policymakers and practitioners

**DOI:** 10.1186/s12966-021-01082-2

**Published:** 2021-02-10

**Authors:** James Nobles, Carolyn Summerbell, Tamara Brown, Russell Jago, Theresa Moore

**Affiliations:** 1 The National Institute for Health Research Applied Research Collaboration West (NIHR ARC West) at University Hospitals Bristol National Health Service Foundation Trust, Bristol, UK; 2 Population Health Sciences, Bristol Medical School, University of Bristol, Bristol, UK; 3 Department of Sport and Exercise Sciences, Durham University, Durham, UK; 4 Fuse, NIHR Centre for Translational Research in Public Health, Newcastle upon Tyne, UK; 5 The NIHR ARC North East & North Cumbria (NIHR ARC NENC), Newcastle upon Tyne, UK; 6 Cochrane Vascular, The Usher Institute, University of Edinburgh, Edinburgh, UK; 7 Centre for Exercise, Nutrition and Health Sciences, School for Policy Studies, University of Bristol, Bristol, UK; 8 Methods Support Unit, Editorial and Methods Department, Cochrane, London, UK

**Keywords:** Wider determinants of health, Childhood obesity, Prevention, Action mapping, Whole systems approach, Intervention design

## Abstract

**Background:**

Randomised controlled trials (RCTs) are often regarded as the gold standard of evidence, and subsequently go on to inform policymaking. Cochrane Reviews synthesise this type of evidence to create recommendations for practice, policy, and future research. Here, we critically appraise the RCTs included in the childhood obesity prevention Cochrane Review to understand the focus of these interventions when examined through a wider determinants of health (WDoH) lens.

**Methods:**

We conducted a secondary analysis of the interventions included in the Cochrane Review on “Interventions for Preventing Obesity in Children”, published since 1993. All 153 RCTs were independently coded by two authors against the WDoH model using an adaptive framework synthesis approach. We used aspects of the Action Mapping Tool from Public Health England to facilitate our coding and to visualise our findings against the 226 perceived causes of obesity.

**Results:**

The proportion of interventions which targeted downstream (e.g. individual and family behaviours) as opposed to upstream (e.g. infrastructure, environmental, policy) determinants has not changed over time (from 1993 to 2015), with most intervention efforts (57.9%) aiming to change individual lifestyle factors via education-based approaches. Almost half of the interventions (45%) targeted two or more levels of the WDoH. Where interventions targeted some of the wider determinants, this was often achieved via upskilling teachers to deliver educational content to children. No notable difference in design or implementation was observed between interventions targeting children of varying ages (0–5 years, 6–12 years, 13–18 years).

**Conclusions:**

This study highlights that interventions, evaluated via RCTs, have persisted to focus on downstream, individualistic determinants of obesity over the last 25 years, despite the step change in our understanding of its complex aetiology. We hope that the findings from our analysis will challenge research funders, researchers, policymakers and practitioners to reflect upon, and critique, the evidence-based paradigm in which we operate, and call for a shift in focus of new evidence which better accounts for the complexity of obesity.

**Supplementary Information:**

The online version contains supplementary material available at 10.1186/s12966-021-01082-2.

## Introduction

The prevalence of childhood obesity has grown rapidly across the world in the last four decades [1]. Throughout the same time, our understanding of obesity has evolved; it is now widely agreed that it is the product of a complex adaptive system with a number of upstream (i.e. government policies and wider economic factors), midstream (e.g. employment, housing & education), and downstream (i.e. behavioural) determinants [2, 3]. Moreover, population health is said to be borne out of the wider conditions in which we are born, live, work and play [4–7]. In turn, this means that stark health inequalities exist between those who live in areas of differing deprivation [6]. Exemplifying this is the prevalence of childhood obesity in England, which in the most deprived areas is double that of the least deprived^1^ [8]; trends which are mirrored in other high-income countries [9, 10]. It is therefore reasonable to assume that the efforts to prevent childhood obesity would account for its complex aetiology and target the wider determinants of health. We set out to test this hypothesis by evaluating the focus of interventions that aim to prevent childhood obesity which have been evaluated in randomised controlled trials (RCTs).

An Action Mapping Tool, which uses the Wider Determinants of Health (WDoH) model [4], was published by Public Health England as part of their Whole Systems Obesity programme [11]. This tool was initially designed to help local authorities in England systematically document their actions on obesity, and to critically reflect upon these in light of the WDoH. Nobles et al. [12] used this tool to explore how 10 local authorities aimed to address obesity and identified that almost two thirds of action aimed to change individual lifestyle factors such as diet and physical activity. Seldom did local authorities aim to address the upstream determinants of obesity, despite the Action Mapping Tool illustrating that 60% of the causes reside upstream. These insights clearly delineate the imbalance between where intervention efforts are placed in contrast to the causes of obesity. The authors suggested that this disparity may be due to an evidence base that is skewed towards downstream interventions, which public health professionals then use to guide their practice.

Randomised control trials are widely regarded as the highest quality source of evidence, and as such, are often used by policymakers and practitioners to shape their planning and decision making [13, 14]. The Cochrane Collaboration builds upon this premise and seeks to synthesise the findings from high-quality RCTs. The findings from the Cochrane Reviews are then regularly used to inform national and international health policy and decision making [15–19]. As such, the focus of the interventions included in the Cochrane Review – and the recommendations stemming from these reviews – will subsequently impact upon the type of interventions which are implemented in the real world. It is important to note here that we recognise that other study designs may be used to evaluate obesity prevention efforts (e.g. natural experiments), but it is the influence of, and the reliance upon, the RCT for policymaking that leads our study to place them at the centre of inquiry.

Since the original publication of the Cochrane Review of interventions aiming to prevent obesity in children in 2001, it has been updated three times in order to keep abreast of the burgeoning evidence base in this field [20–23]. The original version included 10 studies [23], the 2005 version included 22 studies [21], the 2011 version included 55 studies [22], and the latest version in 2019 included 153 studies [20]. Whilst the most recent review [20] did assess the delivery setting of the interventions, it did not map the interventions within the obesogenic system or through a WDoH lens. However, we know that to prevent obesity at the population level, interventions and policies should account for the WDoH [3, 6, 7, 24].

The aim of this study was to understand the extent to which the interventions included in the Cochrane Review on preventing childhood obesity have changed their focus overtime (from 1993 to 2015) when appraised through a WDoH lens. We also aimed to explore whether there was any variation in the intervention focus of studies that targeted children of different ages (0–5 years, 6–12 years, and 13–18 years). We adapted the Action Mapping Tool [11] to analyse the interventions and investigate our research aims.

## Methods

### Study design and data sources

The present study is a secondary analysis of the 153 RCTs contained within the recent Cochrane Review “Interventions for Preventing Obesity in Children” [20]. Given that Cochrane Reviews examine literature internationally, we took these studies to be representative of the global efforts being researched to prevent childhood obesity via RCT design. Several authors included in this study are authors of the Cochrane Review [20]. Ethical approval was not required.

### Action mapping tool

The Action Mapping Tool [11] was used as an analytical framework. This tool was initially designed to allow local authorities to systematically collate information about their actions on obesity. For example, the tool encourages users to provide a thorough description of the action, the metrics used to evaluate this action (e.g. key performance indicators), the organisation(s) responsible for implementing the action, and the extent to which the action aligns with others. It also asks users to state the level of the WDoH model that the action is targeting: i) Biological Factors (BF), ii) Individual Lifestyle Factors (ILF), iii) Social and Community Factors (SCF), iv) Living and Working Conditions (LWC), and v) Wider Conditions (WC). For the purpose of the present study, we used the tool to code the 153 interventions included in the recent Cochrane Review [20] against the WDoH (see data analysis section). This method was similar to the approach applied by Nobles et al. [12] when analysing the efforts of local authorities in England [12]. The tool also enabled us to contrast the causes of obesity against the actions to address obesity; we then present data visualisations of the interventions mapped against the five levels of the WDoH model. This tool, and the subsequent analysis, does not however enable users make claims as to the effectiveness or reach of a given intervention.

### Data analysis

We used an adapted framework synthesis method [25] to guide our analysis, meaning that qualitative data are both quantified and broadly described. This analytical method is consistent with that used in comparable studies [12, 26, 27]. Coding was undertaken using the text available in the “Characteristics of Included Studies” table within the Cochrane Review [20], primarily based upon intervention descriptions. If insufficient data were available in the intervention description, the full text published articles were accessed.

Interventions were coded against the five levels of the WDoH model to account for the three research aims. To address the first research aim, we considered the question: “What and/or who does this intervention aim to change?”. Definitions for each level of the WDoH were used to code the descriptions of the interventions (Table 1). Given that many studies include complex interventions (i.e. are made up of many component parts and target several settings / groups simultaneously [28]), they often had several foci, and as such, each intervention could be coded against one or more of the five levels of the WDoH model. We completed the coding of interventions in three waves to allow discussion and development of congruence in the coding decisions. In the first wave, we applied the WDoH coding framework to 24 studies, with two authors (JN & THMM) independently coding all interventions and then meeting to discuss any coding discrepancies until consensus was reached. We repeated this process for a further 33 studies (i.e. wave 2) and then finally for the remaining 96 studies (wave 3).

**Table 1:** WDoH Coding Descriptions

Level	Description
**Biological Factors (BF)** i.e. the influence of genetic make-up.	**Biological Factors** relate to the genetic factors which people are born with – including sex, ethnicity, age and hereditary factors. Interventions often seek to change the physiological functioning of individuals (e.g. pharmacotherapy, metabolic and bariatric surgery).
**Individual Lifestyle Factors (ILF)** i.e. the influence of our behaviours.	**Individual Lifestyle Factors** relate to behaviours undertaken by individuals and/or their parents/immediate family – for example, smoking, drinking alcohol, dietary consumption, and being active. Interventions often seek to modify the behaviours that individuals, parents or the immediate family make – often through educational programmes, awareness raising (e.g. weight management programmes, behaviour change interventions, physical activity sessions).
**Social and Community Factors (SCF)** i.e. the influence from the people around us.	**Social and Community Factors** relate to the influence of our relationships with those around us (excluding immediate family) on an individual’s health status or their lifestyle behaviours – including neighbours, school friends, work colleagues, faith groups and other social groups. Interventions often seek to change the characteristics and norms of social networks (e.g. training influential peers, increasing capacity within local communities around health-promotion).
**Living and Working Conditions (LWC)** i.e. the influence of where people live, work, and age.	**Living and Working Conditions** are conditions that people spend their daily lives within – for example, their work environments, the quality of their housing, access to education and training, transportation options and links, and their access to good health care. Interventions often seek to change the health offering of peoples’ living and working conditions / environments (e.g. improving the school environment around mental health, incentivising fast food outlets to improve the health-content of their food, improving healthcare professional knowledge around obesity, amending working hours).
**Wider Conditions (WC)** i.e. the influence of the conditions that govern our daily lives.	**Wider Conditions** are the broader socioeconomic, cultural and environmental factors – including how land is used, general levels of disposable income, taxation, and wider areas of political governance. Interventions often seek to change the structures and policies that impact the places in which people live, work, and age *at a societal level*. For example, subsidising sustainable transport options, increasing access to good quality, affordable homes, legislating around fast food marketing to children, and policies against high calorie, low nutrient food and drink.

With regards to the second aim, a more detailed level of coding was applied to understand specifically what, within each level of the WDoH model, the intervention was aiming to change. For example, Alkon et al. [29] was seen to target two levels of WDoH (ILF and LWC), but specifically, the intervention wanted to a) increase parental knowledge on how to improve family health behaviours (ILF), b) upskill catering staff and administrative staff (LWC), c) provide alternative food or drink within the school (LWC), and d) implement a school wide policy reform (LWC). Two authors completed this additional level of analysis, with any disagreements or uncertainties being discussed until consensus reached. We answered the third research aim of this study using the coding applied to address aims one and two.

## Results

### Overview of included studies

Of the 153 RCTs, 39 (25%) included children aged 0–5 years, 85 (56%) included children aged 6 to 12 years, and 29 (19%) included children and young people aged 13–18 years. Most studies were conducted in North America (*n* = 77, 50%) and Europe (*n* = 45, 29%), with relatively few in Australasia (*n* = 15, 10%), Asia (*n* = 7, 5%), South America (*n* = 6, 4%),and the Middle East and North Africa (*n* = 3, 2%). The majority were carried out in high-income countries (*n* = 139; 91%), 13 (8%) in upper-middle-income countries, and one (1%) in a lower-middle-income country (based on the World Bank classifications). Nineteen studies (12%) had a specified aim of targeting children who lived in disadvantaged areas, all of which were in high-income countries. Most (*n* = 91, 59%) were delivered in a school setting (primary, middle and secondary schools); 23 (15%) were delivered in the community (e.g. community centres and venues, local shops, summer camp, fitness centres, the home); 6 (4%) were delivered in a health care setting; 22 (14%) in childcare which included nurseries, child-care centres, kindergartens and pre-schools; and 11 (7%) were delivered in home. The types of intervention in the Cochrane Review [20] were divided into three, those that primarily delivered dietary interventions (*n* = 21, 14%); those in which the intervention was predominantly physical activity (*n* = 39, 21%); and the majority, in which both diet and physical activity were delivered (*n* = 93, 61%). These data were drawn from the Cochrane Review [20].

### Distribution of interventions against the WDoH

**Fig. 1:**
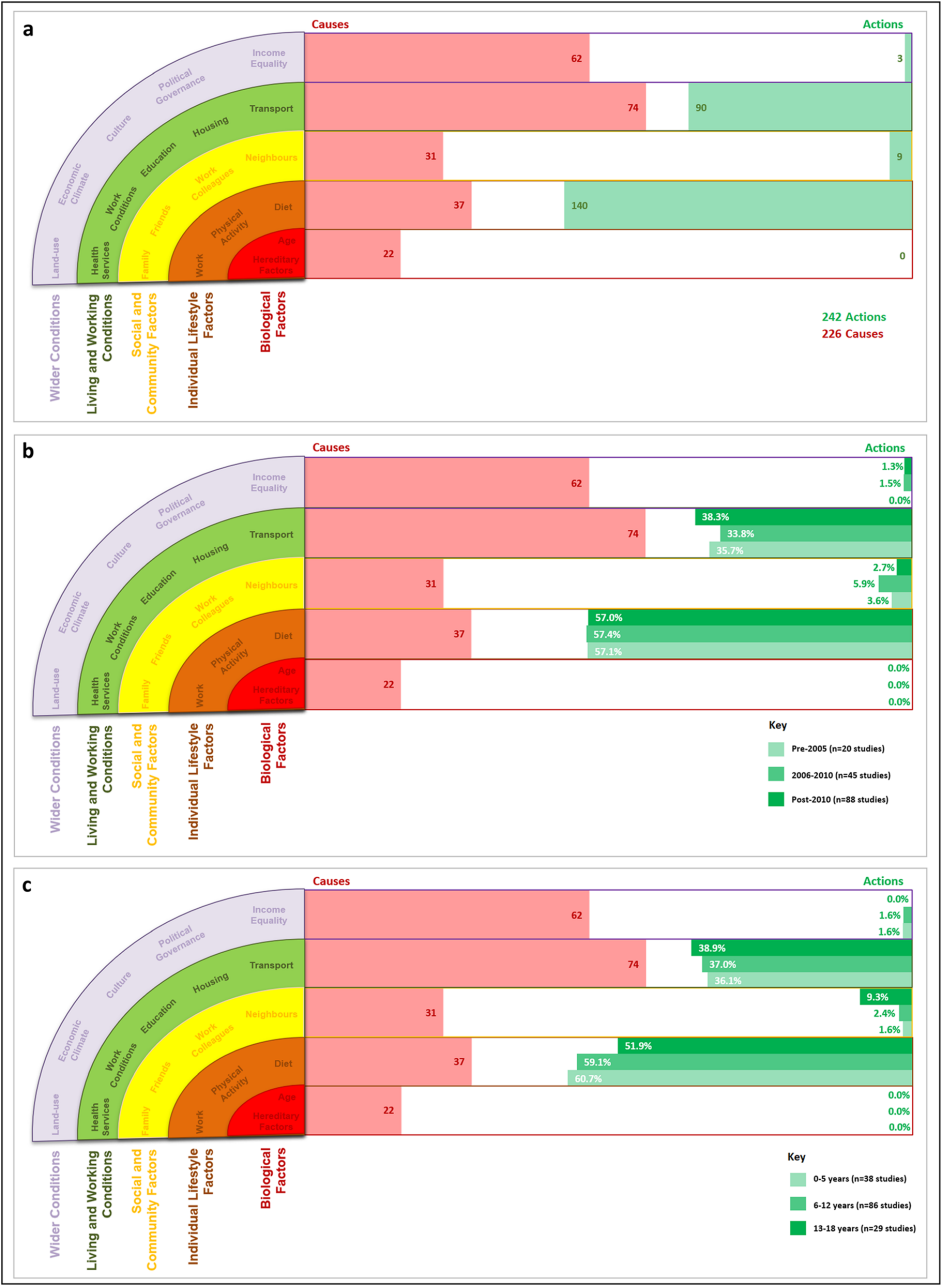
Distribution of intervention efforts against the perceived causes of obesity in the context of the WDoH. Panel **a** represents the distribution of 242 intervention efforts from the 153 studies against the WDoH. Panel **b** presents the same data as panel **a** but separated by the date of publication. Panel **c** presents the same data as panel **a** but separated by the age of the children targeted by the interventions

When looking at the distribution of the 242 intervention efforts from the 153 studies (Fig. 1, Panel a), 57.9% (*n* = 140) of all efforts targeted ILF, 37.1% (*n* = 90) at LWC, 3.7% (*n* = 9) at SCF, and 1.2% (*n* = 3) at WC. None of the interventions sought to change determinants at the BF level. In Fig. 1 (Panel A), the intervention efforts are contrasted against the 226 perceived causes of obesity from Public Health England Action Mapping Tool [11]. Over 60% of causes were coded as LWC (*n* = 74, 32.7%) or WC (*n* = 62, 27.4%), and the remaining 40% were coded as BF (*n* = 22, 9.7%), ILF (*n* = 37, 16.4%) or SCF (*n* = 31, 13.7%) – illustrating a notable imbalance between intervention efforts and the perceived causes of obesity.

Table 2 demonstrates how the interventions focused on multiple WDoH levels. Of those interventions which focused on one level of the WDoH only (*n* = 73), most sought to influence ILF (*n* = 61). Studies that targeted two levels of the WDoH were likely to focus on ILF *and* LWC combined (*n* = 70/74 studies). Seven studies targeted three levels (often ILF, LWC, and SCF), and only one study [30] addressed four levels of the WDoH (ILF, SCF, LWC, and WC respectively). Of note, 140 of the 153 studies (91.5%) had a focus on the ILF level.

**Table 2:** Foci of interventions across multiple levels of the WDoH

Number of WDoH levels interventions coded at^**a**^	Level of the WDoH model					Number of studies (%)
	***BF***	***ILF***	***SCF***	***LWC***	***WC***	
One	–	✓	–	–	–	61 (39.8%)
	–	–	–	✓	–	11 (7.2%)
	–	–	–	–	✓	1 (0.6%)
Two	–	✓	–	✓	–	70 (45.8%)
	–	–	✓	✓	–	1 (0.6%)
	–	✓	✓	–	–	1 (0.6%)
Three	–	✓	✓	✓	–	6 (3.9%)
	–	✓	–	✓	✓	1 (0.6%)
Four	–	✓	✓	✓	✓	1 (0.6%)

^a^Interventions were able to be coded at more than one level of the WDoH model. Table 2 demonstrates the foci of the interventions included within the 153 studies. For example, 61 studies solely focused on changing ILF, whereas 70 studies targeted ILF and LWC combined. These data are based upon the coding completed by two members of the research team (JN and THMM)

### Specific foci of intervention efforts within the WDoH

In the secondary level of analysis, this study sought to understand the focus of intervention efforts *within* each of the WDoH levels. Forty-two codes (i.e. intervention focal points) were agreed upon, of which the 153 studies were coded against on 411 occasions (Additional file 1: Online Supplement I). Studies ranged from one intervention focus (*n* = 39 studies) through to nine (*n* = 1 study), with a mode of two foci per study.

Fourteen codes were generated for interventions targeting ILF. Thirteen of these had education at their core, however seven codes were specifically related to education targeted at parents, and five targeted children. For parents and children alike, the main focus of education was to improve multiple health behaviours (i.e. diet, physical activity, and sedentary behaviour combined) (cited in 55 studies which provided education for parents and in 75 studies for children). Beyond this, educational content varied greatly between studies, from a focus on parenting skills (*n* = 5 studies), to screen time (*n* = 4 studies), to infant feeding (*n* = 4 studies). The one code at the ILF level which was not education-based was the provision of additional after-school physical activity (*n* = 27 studies).

Very few interventions aimed to influence determinants at the SCF level (*n* = 9 studies), with substantial variation between interventions as where efforts were placed. Three interventions sought to influence the social norms around physical activity, with two also targeting social norms around health more broadly. Other efforts included the organisation of social events, community involvement in the intervention delivery, and peer champion training or peer involvement. The level of information provided within study descriptions that pointed towards SCF, in contrast to other levels, was often limited.

For interventions which intervened at the LWC level, consistent patterns emerged within the data, despite 19 codes being created (see Additional file 1: Online Supplement I). The provision of teacher training was the most frequently observed code (*n* = 39 studies), which often meant that interventions aimed to upskill staff so that they can deliver intervention educational material to children. Further structural changes were also noted within the curriculum; 31 interventions provided additional physical activity during school hours and 30 interventions embedded further content about positive health behaviours. Other commonly noted codes included modifications to the food- (*n* = 10 studies) and physical activity- (*n* = 8 studies) environments, as well as the provision of alternative food and drink options largely in school-based settings (*n* = 20 studies).

Three interventions targeted efforts at the WC level. Only two codes were generated for efforts at this level. Two interventions employed state and district wide policies, both of which aimed to influence school food environments. One intervention worked with the Ministry for Public Education to encourage the school level adoption of the obesity prevention programme. This study, by Shamah Levy et al. [31], was considered to be the most comprehensive intervention included within the analysis, and targeted the ILF, LWC and WC levels.

### Changes in intervention focus over time

The pattern of intervention efforts remained consistent over time (see Panel b, Fig. 1). Of the 20 studies published before 2005, 57.1% of intervention effort was placed on ILF and 35.7% on LWC. Between 2006 and 2010, when a further 45 studies were published, these new interventions continued to focus their efforts on changing ILF (57.4%) and LWC (33.8%). With a further 88 studies published after 2010, the focus remained consistent; 57% of intervention efforts focused on changing ILF and 38.3% on LWC. Interventions frequently focused on, and were coded at, more than one level of the WDoH.

### Changes in intervention focus between age groups

Panel c (Fig. 1) highlights that there are no discernible trends between the focus of interventions efforts regarding age groups (< 5 years, 6–12 years, and 13–18 years). One point to note is that slightly more emphasis was placed on changing SCF in interventions developed for 13–18 year olds, often by aiming to change social norms within the cohort (*n* = 3 of the 5 studies targeting SCF). As aforementioned, interventions could be coded at multiple levels.

## Discussion

### Summary of results

This secondary analysis reveals that the majority of the RCT evidence is focused upon the downstream determinants of obesity. This skewed focus has endured since the early 1990s and persists despite the growing recognition that population-level obesity is a complex systems issue. These findings are pertinent because Cochrane Reviews are highly cited and are regularly used to inform local, national and international policymaking [15–19]. Our results suggest that, of the 153 studies included in the recent Cochrane Review, most interventions efforts targeted individual lifestyle factors (ILF; via education on health behaviours) and the living and working conditions (LWC; mainly via teacher training and curriculum changes). Of note, where teacher training was provided (i.e. LWC level), it often aimed to provide teachers with the knowledge and skills to educate pupils about health-related behaviours, again driving action at the ILF level. Over half of the studies targeted more than one level of the WDoH, most of which included ILF and either LWC and/or SCF. We also noted that the focus of interventions has not changed greatly over time, despite demonstrating limited effectiveness in preventing childhood obesity [20]. When looking at the distribution of interventions amongst children of different ages, the approach seems to be somewhat consistent, and most are implemented in early-years centres and schools. Thus, the evidence base in childhood obesity prevention is skewed towards interventions that aim to provide education on how to improve ILF such as physical activity and diet.

### Comparison with existing literature

Using the same approach to analysing the actions of local authorities in England, Nobles et al. [12] found a similar distribution of intervention efforts against the WDoH. The study suggested that nearly 60% of the 280 actions analysed attempted to change ILF, with a dominant focus on education via weight management programmes and general health improvement programmes. The collective findings suggest that the current efforts of local authorities in England, and the RCT evidence base upon which many decisions are made, predominantly rely on individual agency, and assume that via education, individuals can change their behaviours. As a population-level approach to prevention, this is unlikely to be effective [32], and may indeed have negative consequences for health inequalities [13, 24, 32–36]. This is particularly concerning as only 19 studies had a stated aim of targeting children from deprived areas. Our findings are therefore paradoxical given the widespread agreement that upstream efforts are required to alter the obesogenic systems in which we live [2, 24, 34, 37]. This secondary analysis and the study of Nobles et al. [12] provide little evidence to suggest research and practice is moving upstream with regards to obesity prevention. A paradigm shift is needed within the field.

It is important to recognise that there are practical challenges that policymakers face when identifying approaches to implement. Policymakers are pressed to ensure that their proposed strategies are informed by good quality evidence, align with the political direction, are easily measurable / quantifiable, able to demonstrate quick wins, and thus, are perceived to be an appropriate use of public money [38–41]. If the evidence base is focussed on downstream determinants, it is somewhat inevitable that this type of strategy will be implemented. By the same token, researchers working within health (and public health) are often pressed to generate high-quality evidence which demonstrates the efficacy, and effectiveness, of a given intervention [42–44]. This often means that researchers work within a medicalised paradigm, which focuses on the alteration of specific parts of the system in isolation. These interventions can be evaluated via feasibility studies, and subsequently, in adequately powered RCTs [45]. Such interventions are anticipated to work in a predictable and linear manner, so that when they are scaled up, they provide reliable outcomes under real-world conditions. Again, this is known not to be the case due to the complexity of obesity [13, 44–46]. And herein lies a further challenge. Research funding structures are often organised in a way which favour RCTs, and as Rutter et al. [13] noted in the context of the UK NIHR Public Health Research Programme, more than 75% of funded projects tested down- to mid-stream interventions. If research funding generally supports a medicalised model of health, and if policymaking remains tied to having a substantive evidence-base, then a paradigm shift seems unlikely.

In order to move beyond this impasse, alternative research designs (as noted within the introduction) may be required to evaluate multifaceted approaches that function within a complex adaptive system [13, 36, 47–50]. Signs of this are starting to emerge. Two reviews have synthesised childhood obesity prevention efforts which were evaluated through natural experimental designs, both of which include 33 studies [36, 51]. Karacabeyli et al. [36] synthesised *complex* community-based interventions and concluded that interventions which target a wide range of determinants across multiple levels of the WDoH were more likely to be effective than single-sector / setting interventions. Other studies concur [51, 52]. But despite the two reviews focusing on non-RCT design studies, the evaluated interventions (which include policy changes) were typically downstream efforts implemented within schools (Karacabeyli et al. [36] = 29/33 studies, and Bramante et al. [51] = 24/33 studies). Whilst schools are a clear setting for intervention given the amount of time children spend there and their importance within society [52, 53], other aspects of the WDoH need to be considered. At present, it appears that the evidence base (RCT or non-RCT) is largely wedded to the evaluation of downstream intervention. If we wish to see a change in how obesity is addressed in policy and practice, a paradigm shift in the design and evaluation of obesity prevention efforts is imperative [13, 14].

### Implications for policy, practice and research

For those working in policy and practice, our study illustrates a clear skew in the distribution of the RCT evidence base regarding the prevention of childhood obesity. This skew is at odds with the distribution of the perceived causes of obesity. It is important to recognise this imbalance and the implications that it may have for local- and national- policymaking. We hope that this analysis prompts policymakers and practitioners to reflect upon the role of the evidence base when designing new policies or approaches. In line with the conclusions of others, the collective findings would advocate that future efforts (research, policy, and practice) target upstream determinants of the obesogenic system [20, 36, 51, 52].

Correspondingly, for researchers, policymakers and practitioners alike, we should be more attuned to the complexity of obesity. Singular interventions (regardless of their complexity), such as those included within our analysis, are unlikely to impact upon the population-levels of overweight and obesity (often assessed via a change in BMI SDS), especially within the short timeframes that they are being measured against (1–3 years) [13, 36]. It would be beneficial for evaluations to consider how they may help alter the obesogenic system, rather than solely focusing on whether it changes the outcome per se [13]. To do so may require researchers to adopt different research designs and methods (see Egan et al. [54]), and for research councils and funders to value and fund alternative study designs (e.g. well-designed natural experiments) to the same degree as RCTs.

### Strengths and limitations

To the best of our knowledge, this is the first study to systematically critique the RCT evidence base on childhood obesity prevention literature through a WDoH lens. Our analysis provides a clear depiction of where obesity prevention research efforts currently focus, and moreover, contrasts these against the perceived causes of obesity. Our previous work with local authorities [12] caused stakeholders to reflect upon their current approach and to question whether they could intervene in alternative parts of the system [55]. We hope that the current study encourages research funders, researchers, policymakers and practitioners, to consider action in alternative parts of the system.

This study does have several limitations. First, our sampling frame was limited to studies included in the recent Cochrane Review on “Interventions to Prevent Obesity in Children” [20]. Although Cochrane Reviews are widely commended for their rigour, this meant that our sample focused on RCTs which may have skewed our analysis towards studies targeting ILF [56], thus impacting on how we were able to answer the research aims. However, the work of Bramante et al. [51] and Karacabeyli et al. [36] – which included studies of natural experimental design – indicated that they too had a similar focus on ILF. Second, the Cochrane Review [20] synthesised studies which were published until June 2015, and since then, an additional 162 studies have been identified. It may therefore be possible that recently published studies may alter the distribution of the findings. Lastly, our study did not attempt to analyse the degree of leverage that each intervention held for systems change. Broadly, we may infer that interventions aiming to address LWC and WC would have wider population reach and may influence the structures within the obesogenic system [24].

## Conclusions

This study found that the evidence base on childhood obesity prevention has remained steadfast in its focus on changing individual lifestyle behaviours since the 1990’s. This comes despite the widespread acknowledgment that obesity is the product of a complex adaptive system. We hope that the findings from our analysis will challenge research funders, researchers, policymakers and practitioners to question their positioning. Users of the evidence base should reflect upon, and critique, the evidence-based paradigm in which we operate, and call for a shift in focus of new evidence which takes a WDoH lens. Funders should use the insights reported in this paper to reconsider the type of research they support, and enable the further development of methodological tools for the design and evaluation of studies which function upstream.

## Supplementary Information

**Additional file 1.** Online Supplement I.

## Data Availability

Not applicable.

## Abbreviations

BF: Biological Factors
ILF: Individual Lifestyle Factors
LWC: Living and Working Conditions
RCT: Randomised Controlled Trial
SCF: Social and Community Factors
WC: Wider Conditions
WDoH: Wider Determinants of Health

## Footnotes

1 In England, deprivation is measured across seven domains referred to as the Indices of Multiple Deprivation (IMD). IMD is a composite measure that draws on seven domains: 1) income, 2) employment, 3) health deprivation and disability, 4) education skills and training, 5) barriers to housing and services, 6) crime, and 7) living environment. These domains represent many of the conditions noted within the WDoH.
