# Supplementary material for: A secondary analysis of the childhood obesity prevention Cochrane Review through a wider determinants of health lens: implications for research funders, researchers, policymakers and practitioners
Source: Int J Behav Nutr Phys Act. 2021 Feb 10;18:22. doi: 10.1186/s12966-021-01082-2 (PMC7874658; doi:10.1186/s12966-021-01082-2)
Supplement: Supplementary file 1 — Additional file 1. Online Supplement I. [file 12966_2021_1082_MOESM1_ESM.docx]

|  | **Foci points** | **WDoH Level** | **Count** |
| --- | --- | --- | --- |
| 1 | Parent education (improve health behaviours) | ILF | 55 |
| 2 | Parent education (increase physical activity) | ILF | 5 |
| 3 | Parent education (improve diet) | ILF | 14 |
| 4 | Parent education (reduce screen time) | ILF | 3 |
| 5 | Parent education (infant feeding) | ILF | 4 |
| 6 | Parent education (food preparation) | ILF | 2 |
| 7 | Parent education (parenting skills) | ILF | 5 |
| 8 | Child education (improve health behaviours) | ILF | 75 |
| 9 | Child education (improve diet) | ILF | 19 |
| 10 | Child education (increase physical activity) | ILF | 9 |
| 11 | Child education (food preparation) | ILF | 1 |
| 12 | Child education (reduce screen time) | ILF | 1 |
| 13 | Additional PA (non-curricular) | ILF | 27 |
| 14 | App based | ILF | 4 |
| 15 | Catering staff training | LWC | 7 |
| 16 | Administrative staff training | LWC | 1 |
| 17 | Teacher training | LWC | 39 |
| 18 | School nurse training | LWC | 1 |
| 19 | Healthcare professional training | LWC | 1 |
| 20 | Policy change (school) | LWC | 8 |
| 21 | Teacher role modelling | LWC | 3 |
| 22 | Food & drink provision | LWC | 20 |
| 23 | Food & drink provision (home) | LWC | 1 |
| 24 | Curriculum change (extra physical acitvity) | LWC | 31 |
| 25 | Curriculum change (improve diet) | LWC | 2 |
| 26 | Curriculum change (improve health behaviours) | LWC | 30 |
| 27 | Physical activity environment modification | LWC | 8 |
| 28 | Built environment restructure | LWC | 2 |
| 29 | Food environment modification | LWC | 10 |
| 30 | Home environment modifications | LWC | 1 |
| 31 | Advertising / social marketing | LWC | 6 |
| 32 | Additional staff | LWC | 1 |
| 33 | Food & drink substitution | LWC | 2 |
| 34 | Social event | SCF | 1 |
| 35 | Social norms around physical activity | SCF | 3 |
| 36 | Community education (improve health behaviours) | SCF | 1 |
| 37 | Peer involvement | SCF | 1 |
| 38 | Peer champion training | SCF | 1 |
| 39 | Social norms around health | SCF | 2 |
| 40 | Community involvement | SCF | 1 |
| 41 | Influence policy makers | WC | 1 |
| 42 | Area-wide policy (school food assessment) | WC | 2 |
